# Familial Non-Hereditary Gastric Cancer: Diagnosis, Management, Molecular Characteristics and Future Perspective

**DOI:** 10.3390/cancers17193209

**Published:** 2025-10-01

**Authors:** Carlos Pardo, Irina Luzko, Joaquín Castillo-Iturra, Elisa Cantú-Germano, Leticia Moreira

**Affiliations:** 1 Servei de Gastroenterologia, Hospital Clínic de Barcelona, Universitat de Barcelona (UB), c. Villarroel, 170, 08036 Barcelona, Spain; cpardo@clinic.cat (C.P.); luzko@clinic.cat (I.L.); jacastillo@clinic.cat (J.C.-I.); ecantuge7@alumnes.ub.edu (E.C.-G.); 2 Institut d’Investigacions Biomèdiques August Pi i Sunyer (IDIBAPS), c. Villarroel, 170, 08036 Barcelona, Spain; 3 Facultat de Medicina i Ciències de la Salut, Universitat de Barcelona (UB), c. Casanova, 143, 08036 Barcelona, Spain; 4 Centro de Investigación Biomédica en Red de Enfermedades Hepáticas y Digestivas (CIBEREHD), c. Monforte de Lemos, 3-5, 28029 Madrid, Spain

**Keywords:** familial non-hereditary gastric cancer, *H. pylori*, microsatellite instability, epigenetics, polygenic risk

## Abstract

**Simple Summary:**

Gastric cancer is one of the most common and deadly cancers worldwide. While some families carry genetic germline mutations that clearly increase their risk, there are also families with several members affected by stomach cancer but without any known inherited genetic cause. These cases are called familial non-hereditary gastric cancer. This condition is still poorly understood, and there is little guidance on how to identify, follow up, or treat people at risk. Our review aims to describe what is currently known about this gastric cancer risk condition, including possible risk factors, and clinical and molecular features. By gathering and analyzing the available evidence, we hope to help doctors recognize this condition earlier and guide future research. Ultimately, improving our understanding could lead to better prevention, monitoring and treatment strategies for families affected by this form of gastric cancer.

**Abstract:**

Background/Objectives: Gastric cancer (GC) remains a leading cause of cancer mortality worldwide. While most cases are sporadic, approximately 10% show familial clustering with only a minority explained by known hereditary syndromes. The remaining, termed familial non-hereditary gastric cancer (FNHGC), lack a defined high-penetrance germline mutation. This review aims to summarize current knowledge regarding the diagnosis, risk factors, molecular characteristics and management of FNHGC. Methods: A comprehensive narrative review of the literature was conducted focusing on epidemiologic, molecular and clinical studies addressing families with multiple GC cases but no identified germline mutation. Results: The etiology of FNHGC is multifactorial, and *H. pylori*, with its related chronic gastritis, is probably the key driver. Familial clustering likely occurs when combined with other elements such as genetic polymorphisms, shared exposures to risk factors or even epigenetic phenomena. Molecular profiling reveals distinct patterns in familial tumors such as more frequent microsatellite instability; somatic *CDH1* promoter hypermethylation; and recurrent somatic mutations in *TP53*, RHOA and DNA repair genes. Current management focuses on genetic testing to rule out hereditary syndromes, endoscopic surveillance and mitigation of risk factors, with eradication of *H. pylori* paramount. Conclusions: FNHGC represents a distinct subgroup of GC characterized by a multifactorial etiology related to exposure to risk factors and genetic susceptibility although significant gaps remain in fully explaining the condition. Ongoing research holds promise to provide tools for better detection and prevention in order to reduce the burden of GC in familial settings.

## 1. Introduction

**Figure 1:**
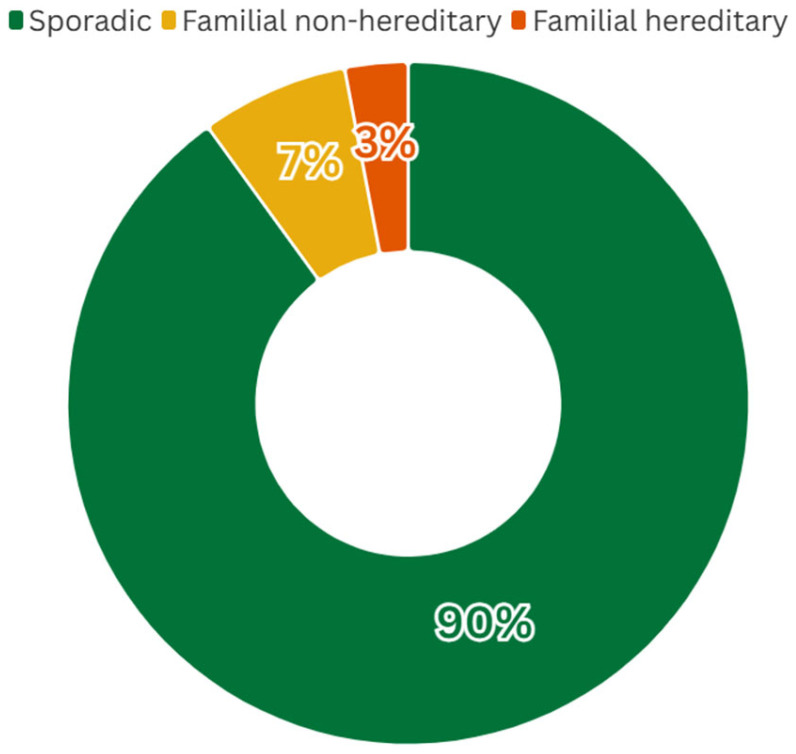
Global distribution of GC cases by category. While ~10% of GCs occur in families with multiple affected members, only ~1–3% of all cases can be attributed to defined hereditary syndromes (caused by known germline mutations). The majority of familial cases have no identified germline cause (“familial non-hereditary”). These proportions are based on published estimates ([2]), and may vary geographically depending on the background incidence of gastric cancer and the prevalence of risk factors such as *H. pylori* infection.

Gastric cancer (GC) is the fifth most common malignancy worldwide and a leading cause of cancer mortality, with an estimated 960,000 new cases and 660,000 deaths annually [1]. Histologically, most gastric adenocarcinomas are classified as either intestinal type (gland-forming tumors) or diffuse type (poorly cohesive, often signet-ring cell carcinoma) according to Lauren’s criteria [2]. Worldwide, the majority of cases are sporadic but around 10% of GC shows familial clustering. Within this familial subset, only a minority (~1–3% of all GC) is caused by known high-penetrance germline mutations (hereditary cancer syndromes) (Figure 1) [3].

Familial non-hereditary gastric cancer (FNHGC) refers to families with multiple GC cases but no identifiable high-penetrance germline mutation. This entity encompasses these unexplained clusters, including familial intestinal gastric cancer (FIGC), an autosomal dominant inherence pattern of intestinal-type GCs but with no known germline mutation, as well as families who meet criteria for hereditary diffuse GC (HDGC) but lack *CDH1* or other known mutations [4].

Since 2000, research has focused on profiling these familial tumors at the molecular level to uncover somatic alterations, gene expression patterns, epigenetic changes and biomarkers that might explain their predisposition or aid in risk assessment. In such families, shared environmental exposures, polygenic traits or undiscovered genetic factors may underlie disease predisposition.

Understanding FNHGC is crucial, as these families may benefit from targeted surveillance or preventive strategies despite lacking an identified genetic marker.

In the present review, we provide an update focusing on FNHGC with the aim of describing the diagnosis, molecular characteristics and implications in surveillance and general management. The discussion of therapeutic interventions or specific treatment strategies lies outside the scope of the present review.

## 2. Materials and Methods

A comprehensive narrative review of the literature was conducted focusing on epidemiological, molecular, and clinical studies related to families with multiple GC cases but no identified germline mutation. Therefore, it does not apply systematic review methodology, and conclusions are based on a qualitative synthesis of the available evidence. Relevant articles were identified using databases such as PubMed and Scopus restricted to studies published in the last 15 years and in English, using the following strings: (“familial”[tiab] or “non-hereditary”[tiab]) and (“gastric cancer”[tiab] or “stomach cancer”[tiab]; hereditary diffuse gastric cancer-like). Articles included were limited to epidemiological, molecular, and clinical studies reporting on families with multiple cases of gastric cancer but without identified high-penetrance germline mutations. Studies focusing on familial clustering of gastric cancer with a known germline mutation were excluded. No formal appraisal of study quality was performed and therefore the certainty of the conclusions should be interpreted with caution.

## 3. Definition and Classification of Familial Gastric Cancer

Familial gastric cancer is generally defined by the occurrence of gastric adenocarcinoma in two or more close relatives. A commonly used criteria is (a) at least 2 first- or second-degree relatives with GC, one of whom was diagnosed before age 50, or (b) at least 3- first or second-degree relatives with GC at any age [5]. Families meeting these criteria account for roughly 1 in 10 GC cases [3]. Within such families, a subset qualifies as hereditary gastric cancer syndromes with Mendelian inheritance, whereas others have no identifiable inherited mutation (the “familial non-hereditary” group of interest).

### 3.1. Hereditary Gastric Cancer Syndromes

Between 1–3% of all GC occurs in the context of a germline mutation that confers an increased risk of developing GC. Clinical suspicion arises in the presence of multiple affected family members, an autosomal dominant inheritance pattern, early age of onset, and the association with other extragastric malignancies. These syndromes are classified into two groups: those that primarily increase the risk of GC and those associated with an elevated risk of both gastric and extragastric neoplasms (see Table 1).

The first group includes HDGC, characterized by an autosomal dominant inheritance pattern and diffuse-type tumors with signet ring cells. Germline mutations in the *CDH1* gene are identified in approximately 30–50% of cases, and less frequently in *CTNNA1*. Another syndrome that primarily increases GC risk is gastric adenocarcinoma and proximal polyposis of the stomach (GAPPS). This syndrome follows an autosomal dominant inheritance with incomplete penetrance and is characterized by fundic gland polyposis with areas of dysplasia or intestinal-type gastric adenocarcinoma confined to the proximal stomach, without evidence of colonic or duodenal polyposis.

The second group comprises hereditary cancer syndromes that confer an increased risk of both gastric and extragastric neoplasms, including Lynch syndrome, Li–Fraumeni syndrome, juvenile polyposis, familial adenomatous polyposis, Peutz–Jeghers syndrome, and hereditary breast and ovarian cancer syndrome (5).

### 3.2. Familial Non-Hereditary Gastric Cancer

As mentioned, after ruling out known hereditary syndromes, any GC occurring in families with multiple affected individuals but without an identified germline mutation can be termed FNHGC. Often these families have later onset, intestinal-type tumors, or do not meet the strict criteria for HDGC. One subset is sometimes called FIGC, defined as an autosomal-dominant familial predisposition to intestinal-type gastric adenocarcinoma with no mutations. Although no single gene has been found to explain FIGC [6], epigenetic alterations have been observed in some cases, hinting at possible underlying mechanisms [3].

#### Summary and Taxonomy of Familial Gastric Cancer Without High-Penetrance Germline Mutations

In conclusion, and as a taxonomy summary we distinguish three partially overlapping entities within the spectrum of familial gastric cancer lacking high-penetrance germline variants. **Familial non-hereditary gastric cancer (FNHGC)** serves as an umbrella term for families with two or more cases of gastric cancer in first- or second-degree relatives, but without identified *CDH1*, *CTNNA1*, or other high-penetrance germline mutations. Within this category, **familial intestinal gastric cancer (FIGC)** refers to families in which affected members predominantly present with intestinal-type gastric cancer, often with an autosomal-dominant inheritance pattern but without a known genetic defect. In parallel, **mutation-negative HDGC-like families** fulfill clinical HDGC criteria but lack identifiable pathogenic *CDH1* or *CTNNA1* variants. Although both FIGC and mutation-negative HDGC-like families fall under the broader FNHGC framework, distinguishing them is important to avoid overgeneralization and to highlight differences in histology, inheritance patterns, and suspected underlying mechanisms. These families are thought to arise from a combination of moderate genetic factors and shared environmental exposures. The followings sections review current knowledge of causes, risk factors, and management strategies for FNHGC.

## 4. Etiology and Risk Factors for FNHGC

Epidemiologic and clinical studies have identified numerous risk factors that increase the likelihood of GC. These risk factors often overlap and compound one another in familial cases. Key risk factors (and their relevance to familial GC) include:

### 4.1. Helicobacter Pylori

This bacterium is the dominant cause of sporadic GC worldwide and is a critical factor in familial clustering of GC. *Helicobacter pylori* (*H. pylori*), a spiral Gram-negative bacterium, colonizes the stomach and induces chronic active gastritis. Over years to decades, this can lead to a precancerous cascade (Correa’s cascade) of histopathologic changes: chronic gastritis, multifocal atrophic gastritis, intestinal metaplasia, dysplasia and culminating in gastric adenocarcinoma. *H. pylori* is classified as a Group 1 carcinogen by the IARC and National Toxicology Program [7]. Importantly, *H. pylori* infection often clusters in families (transmitted in households), thereby providing a plausible explanation for many familial GC cases with no germline mutation [8].

In fact, the combination of *H. pylori* infection and a family history of GC increases the risk. One study showed 5-fold higher odds of GC in *H. pylori*-positive individuals with a first-degree relative affected compared to individuals *H. pylori*-negative and without family history [9]. Families with multiple GC cases have a high prevalence of *H. pylori* infection and resultant chronic gastritis among members [10]. In a landmark prospective trial, eradication of *H. pylori* in asymptomatic carriers reduced subsequent GC incidence by 55% [11]. The benefit was particularly evident in individuals with a family history of GC. Subjects who cleared the infection had dramatically lower cancer rates than those with persistent infection (0.8% vs. 2.9% over 9 years). Thus, *H. pylori* not only initiates the cancer cascade, but also serves as a modifiable risk factor. In a familial context, it is common to find *H. pylori* infection in multiple members. As noted, the combination of *H. pylori* and family history multiplies risk (OR ~5). Therefore, detection and treatment of *H. pylori* in high-risk families is a top priority in risk reduction strategies.

Beyond simply causing inflammation, *H. pylori* may trigger specific molecular changes linked to familial cancer. Notably, chronic infection induces an environment of cytokines (like interleukin-1β, IL-1β) and nitric oxide that can alter gene expression. In vitro studies using gastric epithelial cell lines have demonstrated that *H. pylori* infection can cause epigenetic silencing of tumor-suppressor genes. For example, *H. pylori*–mediated IL-1β release leads to hypermethylation of the *CDH1* (E-cadherin) promoter in gastric cells, with consequent loss of E-cadherin expression [12]. This epigenetic inactivation of *CDH1* in somatic cells mimics the effect of germline *CDH1* mutations seen in HDGC. Such findings reveal how chronic inflammation can “phenocopy” a hereditary mutation via epigenetic mechanisms. In about 17% of familial GC tumors, *CDH1* promoter hypermethylation has been detected, supporting the idea that an inherited propensity for epigenetic changes (perhaps triggered by *H. pylori* or other exposures) could be driving cancer in some familial non-hereditary case [3].

Aside from *H. pylori*, the composition of the gastric microbiome (after *H. pylori*-induced changes) or host epigenetic programming might differ in high-risk families. Research has revealed that chronic *H. pylori* infection can create an “epigenetic field defect” in the stomach, a widespread DNA methylation changes in normal-appearing mucosa [12]. If multiple family members share the infection and perhaps an epigenetically susceptible genetic background, they may all accumulate similar aberrant methylation patterns that predispose to cancer. This area is still being explored. Additionally, concomitant microorganisms (e.g., certain strains of *H. pylori* with the *cagA* virulence factor, or other gastric flora after *H. pylori* eradication) might modulate risk. No specific microbiome signature for familial GC is confirmed yet, but it remains a research interest.

### 4.2. Precursor Lesions (Gastric Atrophy, Intestinal Metaplasia and Dysplasia)

The presence of precancerous lesions in the stomach is associated with an increased risk of GC. Individuals with chronic atrophic gastritis or intestinal metaplasia have a higher likelihood of progressing to cancer over time, especially if the changes are extensive or advanced. First-degree relatives of GC patients tend to harbor more frequent and advanced precursor lesions than age-matched controls. For example, a screening endoscopy study of healthy relatives found gastric intestinal metaplasia in 44% and low-grade dysplasia in 7% of relatives, a markedly higher prevalence than expected in the general population [10]. Another endoscopic surveillance study involving individuals who met the FIGC criteria reported that nearly 50% of patients developed GC precursor lesions during follow-up [13]. This suggests that relatives may develop lesions progressing along the Correa cascade earlier or more extensively (maybe due to shared *H. pylori* strains or genetic susceptibility), putting them on the cusp of malignant transformation. Detecting such lesions via endoscopic screening in relatives can identify those who need closer surveillance or intervention (e.g., endoscopic resection of dysplasia).

### 4.3. Diet and Lifestyle

Dietary habits strongly influence GC risk and often run in families. High intake of salt-preserved foods, smoked/cured meats or fish, and pickled vegetables increases risk, as these foods either contain direct carcinogens (e.g., nitrosamines) or irritate the stomach lining (salt). In regions with familial GC kindreds (e.g., certain areas of East Asia, Latin America), traditional diets high in salt and nitrates are common. Conversely, diets rich in fresh fruits, vegetables, and fiber are protective due to antioxidants and micronutrients. Families often share dietary habits, so a tradition of salt-preserved foods can cause familial aggregation [14,15]. Epidemiologically, migrant studies show that GC risk declines in families adopting diets lower in salt and nitrates over generations.

Low socioeconomic status in childhood, often linked to crowded living (facilitating *H. pylori* spread) and poor diet, is associated with higher GC risk later in life [16,17]. Cigarette smoking is another lifestyle factor: smokers have ~1.5-fold greater risk of GC than never-smokers, with a dose–response relationship. Smoking’s effect is more pronounced for cancers of the gastric cardia, but it also contributes to non-cardia cancer [18]. In familial settings, if the culture of smoking is prevalent, it can add to the baseline risk. Alcohol consumption has a weaker association; heavy alcohol use (particularly hard liquor) may increase risk modestly, though confounding factors make it hard to quantify [19].

### 4.4. Epstein–Barr Virus (EBV)

EBV is associated with ~9% of gastric adenocarcinomas (the EBV-positive molecular subtype) [20]. EBV-positive GCs have a distinct profile of genome-wide promoter hypermethylation [21]. However, EBV-related cases usually occur sporadically; there is no clear pattern of familial EBV-driven GC. Still, EBV is an etiologic factor for a subset of tumors, and co-infection or immunological susceptibility in a family could potentially play a minor role. EBV-associated GCs tend to be proximal and have better prognosis; they might respond to immunotherapy due to abundant viral antigens, which is an emerging consideration.

### 4.5. Host Genetic Susceptibility

It is hypothesized that polygenic factors play a role in familial non-hereditary GC. Certain pro-inflammatory gene polymorphisms that heighten the gastric mucosal response to *H. pylori* have been identified. For example, polymorphisms in the IL-1β gene and related cytokine genes (TNF-α, IL-10) that lead to higher inflammatory cytokine levels are associated with increased GC risk [12]. Individuals who are “high responders” to *H. pylori* (mounting a vigorous inflammatory reaction) develop more extensive atrophy and metaplasia. Such alleles can cluster in families, contributing to familial risk without a single Mendelian mutation. Other low-penetrance genetic factors under investigation include variants in *PSCA*, *PLCE1*, *PRKAA1*, and DNA repair genes identified through genome-wide association studies—their individual impact is small, but in aggregate could influence familial risk [22,23]. For example, certain variants in *PSCA* (prostate stem cell antigen), involved in gastric epithelial cell function, have been linked to GC risk in East Asian genome-wide association studies. Polymorphisms in DNA repair genes (like *XRCC1*) or carcinogen metabolism (*GSTM1* null genotype) have also been studied [24,25]. Each of these polymorphisms confers only a small increase in risk, but if a particular family harbors a cluster of pro-carcinogenic alleles, the combined effect could be significant. Research is ongoing to develop polygenic risk scores for GC; in the future, these might help identify high-risk individuals in familial settings who do not carry a single dominant mutation [26]. Importantly, polygenic risk scores (PRS) should be viewed not as stand-alone predictive tools, but rather as modifiers of environmental and lifestyle exposures within a gene–environment interaction framework. Their clinical value will likely emerge when integrated with risk factors such as *H. pylori* infection, diet, and smoking. Furthermore, caution is warranted regarding the portability of PRS across ancestries, as risk models derived from one population may not directly apply to others without appropriate validation in diverse cohorts.

### 4.6. Autoimmune Gastritis

Pernicious anemia (autoimmune metaplastic atrophic gastritis) leads to achlorhydria and gastric atrophy, conferring a risk of intestinal-type GC. Autoimmune gastritis can run in families (often with other autoimmune disorders). In familial GC cases where *H. pylori* is absent, one should consider autoimmune gastritis as an etiologic factor causing chronic atrophic mucosa prone to neoplastic transformation [27].

In summary, the etiology of FNHGC is multifactorial. *H. pylori* with its ensuing chronic gastritis is probably the key driver, initiating chronic gastritis that, over time, progresses through precancerous stages. Familial cases likely occur when this insult is combined with other factors such as genetic polymorphisms that amplify inflammation or impair DNA repair, shared exposures like carcinogenic diets or smoking, or even epigenetic phenomena, resulting in multiple relatives crossing the threshold to cancer. Unlike hereditary syndromes, no single “cause” can be pinpointed in these families; rather, an interplay of environment and polygenic/modifier genes is responsible. Recognizing these risk factors is important for guiding preventive strategies: for example, test-and-treat for *H. pylori* in relatives, dietary counseling, smoking cessation support, and surveillance of gastric mucosal changes. In the next section, we discuss how these at-risk individuals and families are approached in terms of diagnosis, screening, and management.

## 5. Molecular Characteristics of Familial Gastric Cancer

### 5.1. Somatic Mutation Profiles in FNHGC

Early studies found no obvious driver mutations in germline mutation-negative familial cases. Recent high-throughput analyses have confirmed distinctive somatic landscapes in familial cases. A 2021 study by Carvalho et al. sequenced tumors from 50 FIGC probands from Italy and compared them to sporadic intestinal GCs from a Portuguese cohort. Notably, ~38% (19/50) of FIGC tumors were microsatellite instable (MSI), a significantly higher proportion than in sporadic cases from a comparable geographic cohort. In the same study FIGC tumors also carried a heavier burden of somatic mutations overall and differences in mutational profiles were observed, with differences in key driver genes. For instance, FIGC tumors showed frequent somatic variants in genes like *TP53*, *BRCA2*, *ATM*, *FHIT*, *MSH6*, and *CTNNA1*, whereas sporadic intestinal GCs seldom had *TP53* or *FHIT* alterations. These data suggest familial cases, especially FIGC, may undergo alternate oncogenic pathways, e.g., a “mutator” route via MSI and multiple moderate drivers [26].

Over half (26/50) of FIGC individuals carried common risk-associated variants of *TP53* (polymorphisms) compared to only ~11% (4/38) of sporadic cases. Such variants (e.g., in *TP53*, DNA repair genes, etc.) might subtly alter gene expression or protein function and collaborate to elevate GC risk. Correspondingly, transcriptomic analyses in mouse models (discussed below) suggest that chronic gastric inflammation in a genetically susceptible host induces networks of pro-tumorigenic genes (cytokines, growth factors, cell cycle regulators) distinct from sporadic carcinogenesis. Some candidate gene expression differences in familial GC have been noted. For example, *FHIT*, a tumor suppressor frequently silenced in sporadic GC, was found to have germline and somatic variants uniquely in FIGC tumors, potentially reflecting altered expression or function of this fragile-site gene in familial cases [26].

In diffuse-type familial GCs without *CDH1* mutations, somatic alterations in the E-cadherin pathway are common. Tumors from HDGC families lacking germline *CDH1* mutation have shown E-cadherin inactivation through other mechanisms, for example, loss of heterozygosity or allele-specific downregulation of *CDH1* [28]. Many *CDH1*-mutation–negative diffuse tumors still display reduced E-cadherin protein and *CDH1* expression imbalance, implicating this pathway via somatic hits or epigenetic silencing. Somatic *CDH1* promoter hypermethylation has been observed in ~17% (5/28) of these tumors and *CDH1* loss-of-heterozygosity in ~9%. These data were derived from familial HDGC cohorts and not from direct comparisons with sporadic diffuse gastric cancers. Furthermore, recurrent somatic mutations in genes like *RHOA* (a known diffuse GC driver) appear in both sporadic and familial diffuse GCs. This is exemplified by the finding of somatic *RHOA* mutations in ~25% (22/87) of diffuse GCs overall suggesting that even without an inherited mutation, diffuse familial cases often acquire similar driver events as sporadic tumors [29].

Molecular subtyping efforts such as The Cancer Genome Atlas (TCGA) classification) have revealed that familial GC cases do not form a single distinct subtype, but certain subtypes are enriched [30]. Importantly, familial cases are distributed within the canonical TCGA categories (EBV, MSI, CIN and GS) and do not represent an additional TCGA-level class. As mentioned, FIGC tumors have been reported to be MSI-high in one cohort, aligning many with the MSI subtype of GC which is characterized by high mutation load and promoter methylation in genes like *MLH1*. MSI-H and EBV-positive subsets within familial gastric cancer should be confirmed by standard biomarker assays (e.g., mismatch repair immunohistochemistry, PCR or NGS-based MSI testing, and EBV in situ hybridization). Current recommendations for PD-1–directed immunotherapy in these contexts derive from evidence in unselected gastric cancer populations, as no randomized controlled trials have yet been conducted specifically in familial gastric cancer cohorts. Other cases might fall into the chromosomal instability (CIN) subtype (especially intestinal-type tumors), or the genomically stable subset (diffuse-type tumors). Within diffuse GC-subtype familial cases, many tumors are genomically stable but can harbor the characteristic somatic *RHOA* mutations of that subtype. Familial intestinal tumors, by contrast, tend toward the CIN profile but with the unexpected MSI enrichment noted above. Global gene expression profiling specific to familial GC is limited, but some patterns have emerged. It has been reported that FIGC probands developed cancer ~10 years earlier than sporadic cases and often had a broad spectrum of malignancies in the family (colorectal, breast, etc.), hinting at underlying polygenic factors [26,31].

### 5.2. Epigenetic Alterations in FNHGC

Epigenetic dysregulation is a key focus in families without germline mutations, since inherited epigenetic changes or environment-driven epigenetic “field effects” could underlie cancer risk [32]. One hypothesis was that some familial cases might inherit epimutations, for instance, a silenced allele of a tumor suppressor gene due to promoter hypermethylation in the germline. However, a study of 22 familial/early-onset GC patients found no evidence of germline monoallelic hypermethylation of *CDH1* suggesting that mechanism (which occurs in some Lynch syndrome families) is not a major cause in GC [33]. Nonetheless, epigenetic silencing still occurs somatically, as was already mentioned. Approximately 17% of mutation-negative familial GCs show *CDH1* promoter methylation in the tumor, contributing to E-cadherin loss [3]. Similarly, MSI-high tumors (enriched in FIGC) often harbor widespread CpG island methylation (the CpG island methylator phenotype, CIMP), including hypermethylation of *MLH1* and other genes. This suggests a possible epigenetic susceptibility: families might carry polymorphisms in methylation regulators or have shared exposures that promote methylation.

Beyond DNA methylation, histone modification pathways have been implicated. Notably, a 2015 exome study of HDGC (diffuse GC) families identified a germline variant in *DOT1L*, a histone methyltransferase, as a candidate predisposition gene Donner et al. found a *DOT1L* missense variant (p.Pro1146Leu) in a *CDH1*-negative diffuse GC family, raising the possibility that altered H3K79 methylation could predispose to gastric tumorigenesis [34]. Supporting this, an independent analysis highlighted DOT1L involvement in familial GC: aberrant DOT1L activity was linked to H3K79 methylation changes in tumors from familial cases [32]. Altered histone marks in GC are an active area of research, for example, loss of repressive H3K9me3 correlates with advanced stage and recurrence, and in familial contexts such changes might be more pronounced or occur earlier.

Chronic inflammation in families (e.g., shared *H. pylori* infection or diet) may create an “epigenetic field defect” in the gastric mucosa. Baba et al. (2016) [35] described epigenetic field cancerization in gastrointestinal cancers, whereby even histologically normal mucosa in high-risk individuals accumulates methylation changes predisposing to cancer [35]. In *H. pylori*–affected stomachs, for instance, promoters of tumor suppressors genes like *RASSF1A* and *LOX* often become methylated long before cancer develops [36]. In the familial setting, first-degree relatives of GC patients have been shown to carry elevated methylation in certain genes in their non-neoplastic gastric tissue, hinting that an inherited propensity or common environment (e.g., infection) induces these epigenetic alterations. Additionally, histone acetylation changes tied to *H. pylori* have been observed: the bacterium can promote acetylation of H4 at the p21 gene promoter and upregulate the histone demethylase JMJD2B, thereby activating pro-oncogenic COX-2 expression. Such findings illustrate how an environmental factor shared in families triggers epigenetic shifts that drive gastric carcinogenesis [37].

Finally, non-coding RNAs contribute to the epigenetic and post-transcriptional regulation in familial GC. MicroRNA expression profiles in GC can distinguish tumor subtypes and stages, and familial cases are no exception [38]. A review by Suárez-Arriaga et al. noted microRNAs that regulate key genes implicated in hereditary/familial GC, including *CDH1*, *RHOA*, *CTNNA1*, *INSR*, and TGF-β pathways. For example, miR-9 and miR-10b are known to suppress E-cadherin and have been linked to diffuse GC progression; overexpression of such miRNAs in an individual could phenocopy an E-cadherin mutation by silencing its expression [39]. Though not unique to familial cases, certain miRNA signatures might be enriched in families. Ongoing studies are evaluating circulating miRNAs as non-invasive biomarkers to identify high-risk individuals. A three-miRNA plasma signature (miR-18a, miR-21, miR-421) was proposed as a diagnostic marker for GC if validated, such markers could be especially useful in screening members of mutation-negative GC families, who currently lack a molecular test [40].

### 5.3. Candidate Predisposition Genes

From germline exome sequencing of unexplained families, researchers have proposed new candidate predisposition genes. In a Finnish HDGC family without *CDH1* mutations, Donner et al. identified rare germline variants in *INSR* (insulin receptor), *FBXO24*, and *DOT1L* [33]. While these were not recurrent in other families, *INSR* was highlighted for its link to E-cadherin modulation (insulin signaling can affect E-cadherin glycosylation). Likewise, a Korean study using whole-genome sequencing uncovered a unique germline *RHOA* mutation (p.R129W) segregating in one HDGC family [29]. Functional assays showed this mutant RhoA had increased GTP-binding and perturbed downstream YAP1 signaling, enhancing cell migration—consistent with a pro-carcinogenic effect. Although family-specific, this finding implicates the RhoA/ROCK pathway as a mechanistic driver in gastric carcinogenesis and suggests that in some familial cases, germline variants in signaling genes can confer risk. More recently, Herrera-Pariente et al. [41] identified *CTNND1* as a germline biomarker for the predisposition for early-onset gastric cancers and contributes to the disruption of cell–cell interactions, which can potentiate cell motility and metastasis. Thus, germline genetic variants in *CTNND1* could explain some of the current FNHGC [41].

Key themes include (1) a likely polygenic inheritance of moderate-risk alleles (affecting DNA repair, cell adhesion, etc.), (2) an essential role for inflammation and environment (e.g., *H. pylori* triggers that cause somatic mutations and epigenetic alterations), and (3) convergence on known cancer pathways (such as cell adhesion via E-cadherin, TP53 dysfunction, and signaling via RhoA, β-catenin, etc.) despite the absence of a single germline driver. Ongoing molecular profiling, including whole-genome sequencing, epigenome mapping, and proteomics in familial GC cohorts will further clarify biomarkers of risk and potential preventive targets.

Therefore, families with unexplained GC susceptibility have been challenging to decipher, but two decades of research have uncovered important molecular clues. Familial GC tumors often follow different molecular pathways than truly sporadic cases with a tendency toward high mutation loads (e.g., MSI), multiple co-occurring somatic hits, and involvement of classic GC genes through non-germline means (such as E-cadherin loss via methylation or post-transcriptional repression). While a single causative gene remains elusive for most of these families, a polygenic/epigenetic model is emerging: moderate-risk germline variants in genes controlling cell adhesion, DNA repair, or inflammation combine with shared environmental exposures (like *H. pylori* and diet) to drive gastric carcinogenesis. This model is supported by animal and cell-line studies demonstrating that chronic inflammatory stimuli in a susceptible host can recapitulate the somatic and epigenetic changes seen in familial tumors.

Crucially, the growing list of molecular biomarkers, from *TP53* polymorphisms and DNA methylation patterns to microRNA signatures, offers potential tools for early detection in familial GC kindreds. For instance, endoscopic surveillance might be guided by molecular assays that detect field defects (e.g., methylated marker genes) in gastric biopsies, and blood-based markers (gene expression or miRNA panels) could identify carriers of familial risk. On the treatment front, understanding a tumor’s molecular profile can inform therapy: MSI-high familial tumors might benefit from immunotherapy, and RHOA-activated diffuse tumors might be susceptible to inhibitors of the Rho/ROCK pathway or YAP signaling. Although it should be noted that MSI-high tumors are only a subset of FNHGC and its prevalence varies by region and histology.

In summary, molecular characterization of mutation-negative familial GCs has revealed a tapestry of alterations (genetic, epigenetic, and transcriptomic) that together promote tumor development. Ongoing research and comprehensive profiling (through next-generation sequencing, methylome and proteome analysis) are poised to further unravel the “familial” GCs. These insights will not only improve risk stratification and preventive strategies for high-risk families, but also shed light on gastric carcinogenesis in general, bridging the gap between hereditary and sporadic disease. The ultimate goal is a fuller mechanistic understanding that can drive personalized surveillance.

## 6. Current Approaches to Diagnosis and Management

### Identification and Surveillance of High-Risk Families

**Figure 2:**
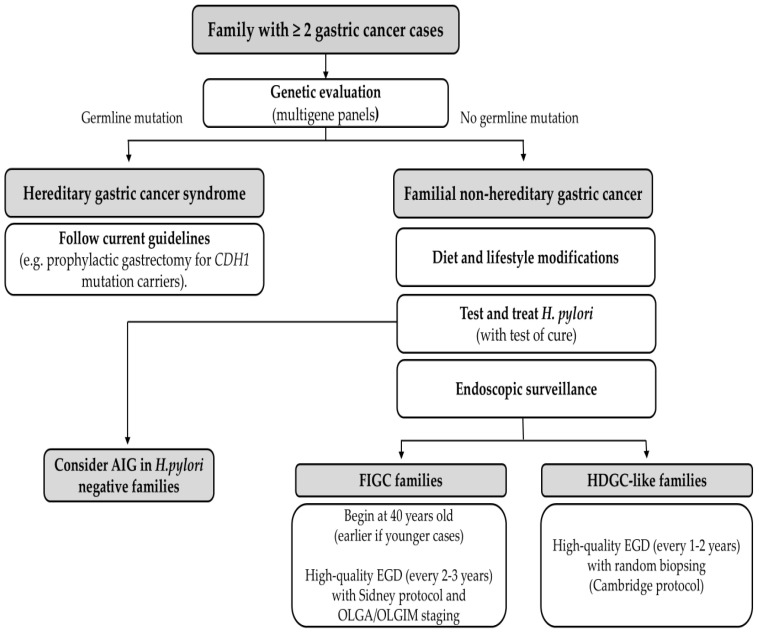
Management algorithm for familial gastric cancer. AIG, autoimmune gastritis; FIGC, Familial Intestinal Gastric Cancer; HDGC-like, Hereditary Diffuse Gastric Cancer-like; OLGA, Operative Link on Gastritis Assessment; OLGIM, Operative Link on Gastritis Intestinal Metaplasia. EGD: esophagogastroduodenoscopy.

Early identification of families with clustering of GC is critical. Clinicians should obtain detailed family histories in any patient diagnosed with GC, noting any relatives with stomach cancer (or related cancers like lobular breast cancer that could suggest HDGC). When multiple cases are present, especially if any occurred at a young age (<50) or are diffuse-type histology, a referral to genetic counseling is indicated to evaluate for hereditary syndromes. Genetic testing (typically via multigene panel) is then performed to look for known germline mutations. Modern GC gene panels include *CDH1*, *CTNNA1*, *APC* (including variants in promotor 1B), *ATM*, *BMPR1A*, *BRCA1*, *BRCA2*, *BRIP1*, *EPCAM* (*E8–E9* deletion), *MLH1*, *MSH2*, *MSH6*, *MUTYH*, *NBN*, *PALB2*, *PMS2*, *PTEN*, *RAD51C*, *SMAD4*, *STK11* and *TP53* [42]. If a pathogenic variant is found, the family is managed according to guidelines for that syndrome (see Figure 2). For example, carriers of *CDH1* mutations are advised to consider prophylactic total gastrectomy typically between ages 20–40, as this is the definitive way to prevent diffuse GC.

However, in the majority of familial GC families, no mutation is identified. These “mutation-negative” families (FNHGC) still have elevated risk, but there is no targeted intervention like prophylactic surgery. The management approach in such cases relies on endoscopic surveillance and risk factor modification. International consensus guidelines (e.g., the International Gastric Cancer Linkage Consortium, IGCLC) recommend that first-degree relatives in familial GC families undergo periodic endoscopic screening. For HDGC-like families who tested negative for *CDH1*, intensive surveillance is advised: high-quality upper endoscopy (esophagogastroduodenoscopy, EGD) every year or two with multiple random biopsies of the stomach, given that early diffuse cancers can be subtle [43]. For families with intestinal-type gastric cancers (FIGC), some experts suggest starting EGD screening around age 40 (earlier if any cases occurred young), every two or three years with biopsies with Sidney protocol and histological evaluation with OLGA and OLGIM staging (in order to rule our preneoplastic lesions) (Figure 2) [44].

A cornerstone of managing any high-risk family is aggressive *H. pylori* detection and treatment. All first-degree relatives of an index GC patient should be tested for *H. pylori* (via urea breath test, stool antigen, or endoscopic biopsy) and treated if positive. This strategy is supported by strong evidence (as was mentioned previously) [11]. Eradication is low-risk and cost-effective in this population. Thus, “test-and-treat” for *H. pylori* in relatives is now recommended by many experts as a preventive measure. Alongside *H. pylori* eradication, relatives should be counseled on dietary and lifestyle modifications: adopt a diet high in fresh produce and low in salt-preserved foods, avoid smoking, moderate alcohol intake, etc.

In summary, diagnostic and preventive management of FNHGC focuses on (1) careful risk assessment (including genetic testing to rule in/out known syndromes), (2) endoscopic monitoring of the stomach, and (3) aggressive mitigation of risk factors (*H. pylori* eradication being paramount).

## 7. Future Perspectives and Research Directions

FNHGC remains an area of active research, as uncovering its underlying causes could significantly improve prevention and early detection. Future efforts are likely to focus on several key areas:

### 7.1. Discovery of New Predisposition Genes

Despite extensive study, the majority of familial GC cases have no identifiable mutation. Ongoing research with next-generation sequencing (whole exome/genome sequencing) in unexplained families may reveal novel rare variants. For example, the discovery of variants in *CTNND1* as a cause of HDGC in a few families suggests other genes in the E-cadherin pathway might be involved [41]. Large international consortia are pooling familial cases to increase power to detect new germline mutations or risk alleles (although external validation in large independent cohorts is still missing). Polygenic risk scores are also being developed by integrating the effects of dozens of single-nucleotide polymorphisms (SNPs) associated with GC. In the future, a polygenic risk score might help identify individuals from moderate-risk families who have a high genetic risk burden and should undergo intensive surveillance, even if no single mutation is present.

### 7.2. Molecular Profiling and Biomarkers

Advances in molecular characterization of gastric tumors (such as The Cancer Genome Atlas classification of GC into subtypes: EBV-positive, MSI, chromosomally unstable, genomically stable) provide insights that could be applied to familial cases. For instance, if familial tumors predominantly show certain molecular features (e.g., particular mutational signatures or methylation patterns), that could point to inherited factors. Epigenomic studies of gastric mucosa from people with a family history (but no cancer yet) is an emerging field, detecting a “methylation fingerprint” of risk could lead to a non-invasive biomarker (for example, a blood or gastric fluid DNA methylation assay) to stratify high-risk individuals. Likewise, microbiome analysis beyond *H. pylori* is being explored; perhaps a synergy of microbial communities contributes to carcinogenesis in some families. Identifying such factors could lead to probiotic or antibiotic interventions to alter the gastric microbiome risk profile.

### 7.3. Preventive Strategies

On the prevention front, a major goal is broad implementation of *H. pylori* eradication in at-risk populations. Japan and other countries are already moving toward population-wide *H. pylori* screening and treatment. For familial cases, one can envision a protocol where as soon as a GC is diagnosed, all first-degree relatives are automatically offered *H. pylori* screening/treatment and endoscopy. Health economic studies indicate this approach is cost-effective, especially in intermediate-to-high incidence settings [45]. Another future possibility is the development of an *H. pylori* vaccine. Research has been ongoing on *H. pylori* vaccine candidates, both prophylactic vaccines to prevent infection and therapeutic vaccines to help clear it. A successful vaccine could dramatically reduce GC globally and would be particularly beneficial for young people in high-risk families, preventing them from ever acquiring the carcinogenic infection in the first place. While no vaccine has completed Phase III trials yet, this remains a critical research direction [46].

### 7.4. Improved Screening and Early Detection

Technological advances in endoscopy and imaging offer promise for earlier and more reliable detection of gastric neoplasia. High-resolution endoscopes with image-enhancement (narrow-band imaging, chromoendoscopy) can highlight subtle mucosal changes. Another burgeoning field is AI-assisted endoscopy: computer vision algorithms can help endoscopists identify tiny lesions or areas of intestinal metaplasia with greater accuracy. This could be especially useful when performing surveillance in familial cases, where the endoscopist needs to thoroughly evaluate a presumably normal stomach for any early signs. Outside of endoscopy, research into non-invasive tests for GC is ongoing: for instance, blood tests detecting circulating tumor DNA, microRNAs, or other biomarkers. If sensitive enough, such tests could be used to screen high-risk family members periodically, with endoscopy reserved for those who test positive.

### 7.5. Interdisciplinary Care and Registries

Moving forward, comprehensive management of familial GC will benefit from specialized centers and registries. Much like high-risk breast/ovarian clinics for *BRCA* families, high-risk GC clinics are beginning to emerge, offering multidisciplinary care (gastroenterologists, geneticists, surgeons, dieticians, psychologists) for these families. International collaboration through registries (like the IGCLC database) will continue to provide valuable data on outcomes of surveillance versus surgery in mutation-negative families, the natural history of precancerous lesions in relatives, etc. These data will refine guidelines, for instance, determining the optimal age to start and stop endoscopic surveillance in various scenarios, or when to escalate to prophylactic surgery even without a known mutation (in exceptionally high-risk lineages).

### 7.6. Emerging Therapeutic Strategies

Recently, cytotoxic peptides with synergistic oncolytic and immunomodulatory effects have emerged as promising candidates for cancer treatment. Several of these peptides have already entered clinical trials for cancer immunotherapy, including gastric cancer [46,47].

## 8. Conclusions

**Figure 3:**
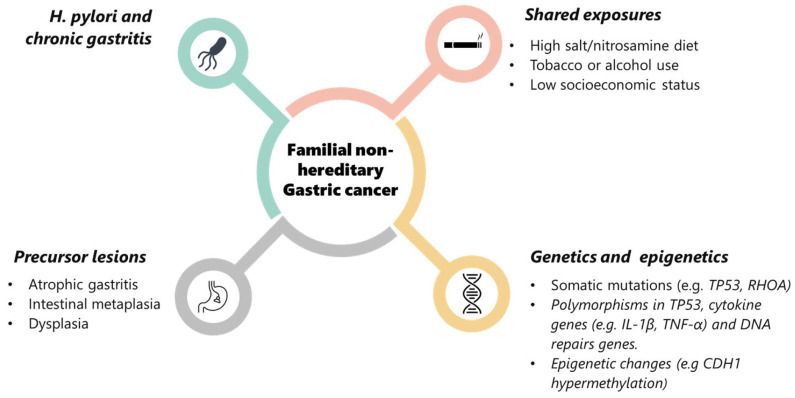
Multifactorial pathogenesis of familial non-hereditary gastric cancer. Schematic diagram of the main contributing factors to FNHGC: *Helicobacter pylori* infection and chronic gastritis, shared environmental exposures (e.g., high-salt/nitrosamine diet, tobacco or alcohol use, low socioeconomic status), precursor lesions (atrophic gastritis, intestinal metaplasia, dysplasia), and genetic/epigenetic alterations (somatic mutations, low-penetrance polymorphisms, and epigenetic changes such as CDH1 hypermethylation). Together, these factors interact to promote gastric carcinogenesis in families without identifiable high-penetrance germline mutations.

FNHGC represents a convergence of risk factors rather than a single causative mutation (see Figure 3). The past two decades have greatly advanced our understanding, from the fundamental role of *H. pylori* and inflammation-driven epigenetic changes to evidence-based interventions like *H. pylori* eradication and endoscopic screening that can save lives. Yet, significant gaps remain in fully explaining why some families are so prone to this disease. The ongoing research into genetic modifiers, epigenetics, and improved detection will hopefully yield new tools to identify at-risk individuals before cancer develops. With earlier identification and tailored prevention, we aspire to further reduce the burden of GC in familial settings, even in the absence of a defined hereditary syndrome. Ultimately, the lessons learned from studying these families may not only benefit them directly but also could possibly shed light on gastric carcinogenesis in general, paving the way for better strategies against this global killer. However, the available evidence remains limited, and conclusions should therefore be interpreted with caution.

## Figures and Tables

**Table 1:** Hereditary gastric cancer syndromes and genes involved.

Hereditary Gastric Cancer Syndrome	Genes Involved
Hereditary diffuse gastric cancer	*CDH1* and *CTNNA1*
GAPPS	*APC* (gene promotor)
Lynch syndrome	*MLH1*, *MSH2*, *MSH6*, *PMS2* and *EPCAM*
Li–Fraumeni syndrome	*TP53*
Juvenile polyposis	*BMPR1A*, *SMAD4*
Familial adenomatous polyposis	*APC*, *MUTYH*
Peutz–Jeghers syndrome	*STK11*
Hereditary breast and ovarian cancer syndrome	*BRCA1*, *BRCA2*, *PALB2* and others

## Data Availability

Not applicable.

## Abbreviations

The following abbreviations are used in this manuscript:

GC	Gastric cancer
FNHGC	Familial non-hereditary gastric cancer
FIGC	Familial intestinal gastric cancer
HDGC	Hereditary diffuse gastric cancer
GAPPS	Gastric adenocarcinoma and proximal polyposis of the stomach
*H. pylori*	*Helicobacter pylori*
EBV	Epstein–Barr Virus
MSI	Microsatellite instability
CIN	Chromosomal instability
SNPs	Single-nucleotide polymorphisms
TCGA	The Cancer Genome Atlas
AIG	Autoimmune gastritis
OLGA	Operative Link on Gastritis Assessment
OLGIM	Operative Link on Gastritis Intestinal Metaplasia
EGD	Esophagogastroduodenoscopy
